# Biomechanical comparison of four triangular osteosynthesis fixations for unilateral vertical sacral fractures

**DOI:** 10.1038/s41598-023-31418-w

**Published:** 2023-03-17

**Authors:** Yupeng Ma, Yong Zhao, Huanyu Hong, Tao Huang, Yu Li

**Affiliations:** 1 Orthopaedics Department, Yantai Shan Hospital, 91#, Jiefang Road, Yantai, 264008 Shandong Province People’s Republic of China; 2 Yantai Key Laboratory for Repair and Reconstruction of Bone & Joint, Yantai Shan Hospital, Yantai, People’s Republic of China

**Keywords:** Fracture repair, Computational biophysics

## Abstract

To compare the stability and biomechanical characteristics of four commonly used triangular osteosynthesis techniques to treat unilateral vertical sacral fractures and provide a clinical application reference. Finite element models of Tile C-type pelvic ring injury (unilateral Denis II sacral fracture) were produced. In four models, sacral fractures were fixed with a combination of unilateral L5, unilateral L4, and L5 iliac lumbar fixation with lengthened or normal sacroiliac screws. The biomechanical properties of the four fixation models were measured and compared under bipedal stance and lumbar rotation. The fixation stability of the model with the lengthened sacroiliac screw was excellent, and the fracture end was stable. The stability of fixation using unilateral L4 and L5 segments was close to that of unilateral L5 segment fixation. Triangular osteosynthesis transverse stabilization devices using lengthened sacroiliac screws can increase the vertical stability of the sacrum after internal fixation and increase the stability of the fracture. When triangular osteosynthesis lumbar fixation segments were selected, simultaneous fixation of L4 and L5 segments versus only L5 segments did not significantly enhance the vertical stability of the sacrum or the stability of the fracture end.

## Introduction

Unstable pelvic fractures arising from high-energy trauma are a challenge for clinical treatment, and surgery is mainly used to re-establish the stability of the pelvic ring. Anatomic repositioning and solid internal fixation of the posterior pelvic ring are the main goals of surgical treatment. The sacrum is an essential part of the posterior pelvic ring, and sacral fractures account for approximately 28–45% of pelvic fractures, of which unstable fractures account for 17–30%^1–3^. Approximately 90% of sacral fractures are accompanied by injuries to other parts of the pelvic ring^4^. The primary goals of surgical treatment of posterior pelvic ring injuries are anatomic repositioning and adequate internal fixation, with additional nerve exploration and decompression for patients with associated neurologic impairment.

A variety of methods for vertically unstable sacral injuries have been advocated, including transiliac rods^5^, transiliac plates^6^, percutaneous sacroiliac screws^7,8^, and spinopelvic instrumentation^9,10^. Advocates of these fixation techniques recommend a similar postoperative rehabilitation programme with partial weight-bearing or prohibition of weight-bearing for 6–12 weeks postoperatively. Previous posterior ring fixation methods are not sufficiently strong to allow for early weight-bearing functional exercise.

Wenning et al.^11^ have conducted comparative studies of lumbopelvic fixation versus iliosacral screw fixation. This study allowed lumbopelvic fixation for early weight-bearing activities in a tolerated situation. The study concluded that lumbopelvic fixation has biomechanical advantages over iliosacral screw fixation. However, some scholars have expressed doubts about the stability of lumbopelvic fixation. Schildhauer et al.^12^ concluded that the iliolumbar fixation method does not maintain the rotational stability of the posterior pelvic ring. Because of its enhanced vertical stability, a 2-point fixation in the vertical direction cannot accomplish rotational stability. This type of fixation does not allow early weight-bearing.He proposed combining a spinal-pelvic fixation system with fixation with sacroiliac screws or sacral plates to treat sacral fractures, called the “triangular osteosynthesis.” Biomechanical analysis confirmed that triangular segmental lumbopelvic instrumentation is the most stable fixation method^12^. Such strong fixation allows patients to do early mobilization and carry out progressive weight-bearing activity post-operation^12–14^.

The most commonly used triangular fixation technique for sacral fractures is iliolumbar fixation combined with sacroiliac screws, and excellent postoperative results have been achieved. However, one literature review revealed few biomechanical studies on triangular fixation techniques. Some questions regarding triangular osteosynthesis remain, as follows: Does using lengthened sacroiliac screws provide better stability of sacral fracture fixation than normal sacroiliac screws? Does the addition of L4 segmental fixation increase the stability of sacral fracture fixation? By clarifying these questions, we can better guide our surgical planning for sacral fractures. This study aimed to create a model of triangular fixation and investigate its biomechanical properties utilizing a 3D finite element method. This study aims to provide a theoretical basis for the clinical use of this technique.

## Methods

This study was approved by the ethics committee of Yantai Shan Hospital and was carried out in accordance with the ethical standards of the Declaration of Helsinki. Informed consent was obtained from each participant included in the study.

### Finite element modelling

**Figure 1:**
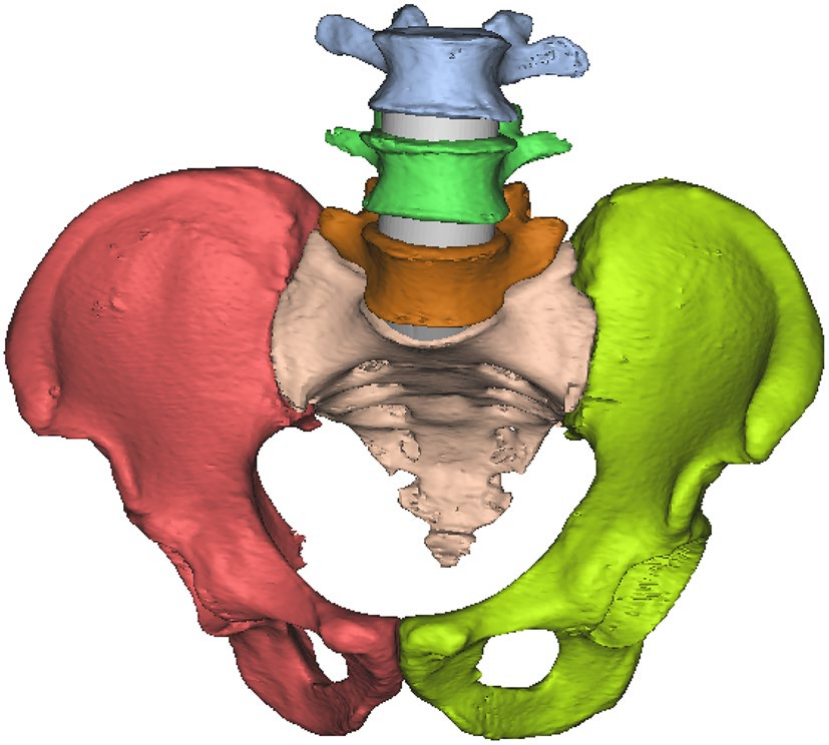
Finite element model generation based on CT data.

This study was based on CT (64-slice spiral CT (Philips)) scan data of L3–L5 and pelvis of a healthy adult female (165 cm, 35 years, 65 kg),The young female consented to her CT-scan was utilised for in the study. The slices were 1 mm thick. A virtual 3D model of the lumbar spine and pelvis was created from the CT data in DICOM format with image processing software (mimics 17.0). The individual components shown in Fig. 1 below were generated based on the CT grey value segmentation technique. The preliminary model of the pelvic spine obtained from MIMICS cannot be used directly for finite element calculations; the 3D model of the pelvis obtained in MIMICS needs to be further processed in the software 3-Matic to make the model smooth for further processing.

**Figure 2:**
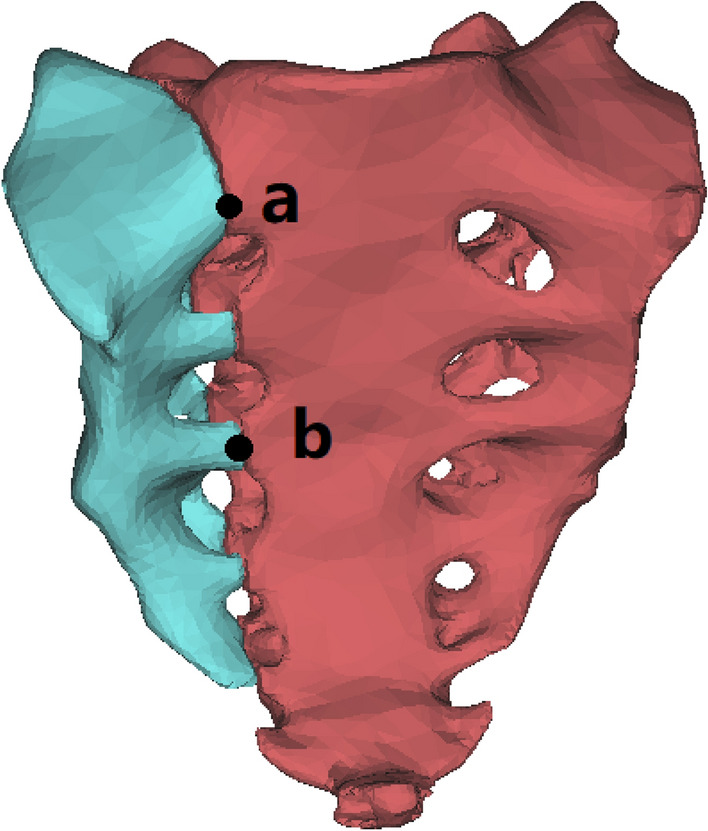
A vertical fracture line was made through the right sacral foramen and points a and b were marked on the fracture line.

The sacral model was incised along the unilateral sacral foramen to simulate fracture of the sacrum. The original single sacrum was divided into two parts to produce a unilateral vertical sacral fracture model (AO type C3.1, Denis II), as depicted in Fig. 2.

The components of the model generated were imported into the 3-Matics Remsh module for meshing. The result of such meshing is a four-node mesh with three degrees of freedom per node. The mesh model of each part was then imported back into Mimics software for assigning material parameters. The material properties of the model were set to non-homogeneous and isotropic material.

The model material was assigned to different skeleton parts using a greyscale-based method. The mimics include a formula to assign ash values to ten levels. The material assignment formula was based on the literature^15^.

The implant was modelled using Solidworks software. It was imported into 3-Matics for pelvic bone model assembly, and meshing was performed. Then, it was imported into mimics for assigning material properties; titanium alloy was used as the implant material.

### Finite element model validation

The finite element model uses spring units to simulate the pelvis and the primary ligament structure around the lumbar spine to ensure the mobility and stress transmission of the sacroiliac joints of the lumbar spine joints. Based on the displacement results of the pelvic model, the maximum mobility of the anterior edge of the sacrum tended to move forwards and downwards, and the iliac bones on both sides tended to rotate in agreement with the literature^16^. The partial lumbar spine movement results were excellent and consistent with the in vitro experimental results.

### Establishment of the ligament and muscle model and the application of load

**Figure 3:**
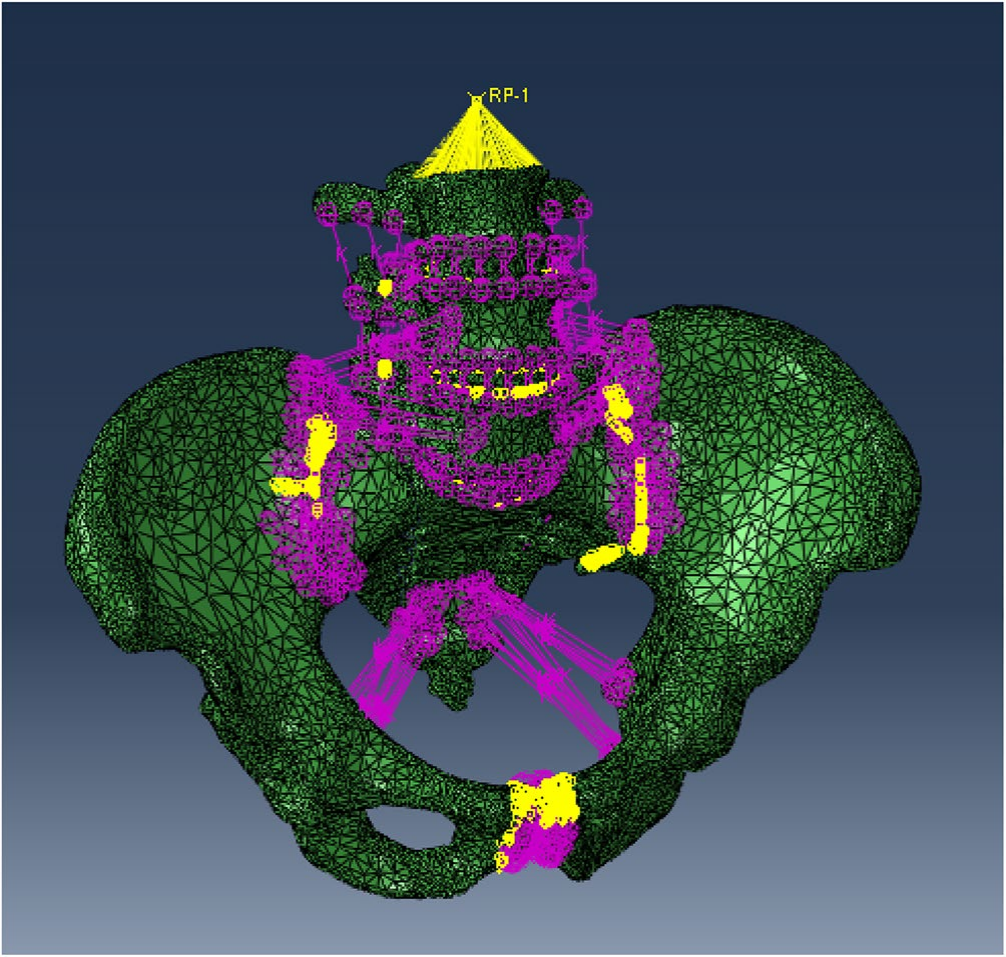
Finite element model after material assignment and ligament linkage.

The mesh models of bones and screws were imported into the software Abaqus, and then spring damping cells were used to simulate the ligaments and muscles. The generated model is illustrated in Fig. 3. The model material parameter settings and ligament parameter settings are shown in Tables 1 and 2^15,17–21^.

**Table 1:** Parameters of the lumbar spine model and implants.

Material	Elastic modulus, MPa	Poisson ratio	Cross-section area, mm^2^
Disc annulus	8.4	0.45	
Disc nucleus	Mooney–Rivlin c1 = 0.12, c2 = 0.03		
Anterior longitudinal ligament	7		63.7
Posterior longitudinal ligament	7		20
Ligamentum flavum	3		40
Intratransverse ligament	7		1.8
Capsular ligament	4		30
Interspinous ligament	6		40
Supraspinous ligament	6.6		30
Implants	11,400	0.3

**Table 2:** Model parameters of pelvic ligaments.

Material	K, N/m	Number of springs
Anterior and capsule sacroiliac ligament	700	27
Posterior sacroiliac ligament	1400	15
Interosseous sacroiliac ligament	2800	8
Iliolumbar ligament	2800	30
Sacrospinous ligament	1400	9
Sacrotuberous ligament	1500	15
Superior pubic ligament	500	24
Arcuate pubic ligament	500	24

The sacroiliac joint and pubic symphysis were set as bound constraints. Six degrees of freedom constraint was performed at the bilateral acetabular nodes. A force of 600 N was applied vertically downwards onto the surface of the upper endplate of L3 to simulate the human body under its gravity when standing upright. A 100 N of slave load and 7 Nm of torque were applied to the upper endplate of L3 around the mechanical axis of the spine to simulate the forces acting on the lumbar rotation.

**Figure 4:**
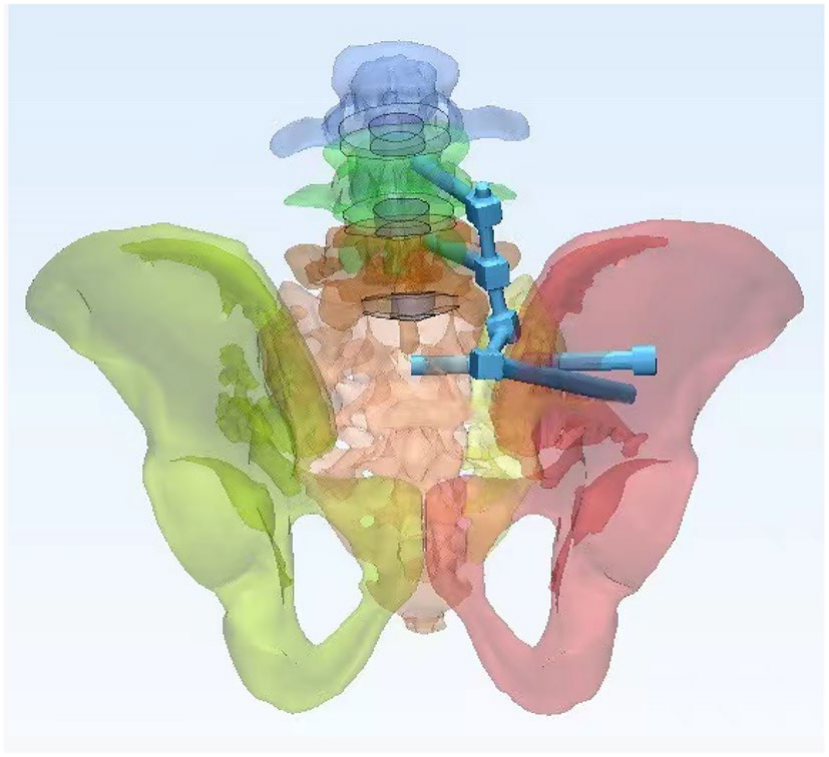
Sketch map of L4L5NS1 (created by Mimics17.0 https://www.materialise.com/en/healthcare/electronic-instructions-for-use/mimics-research).

**Figure 5:**
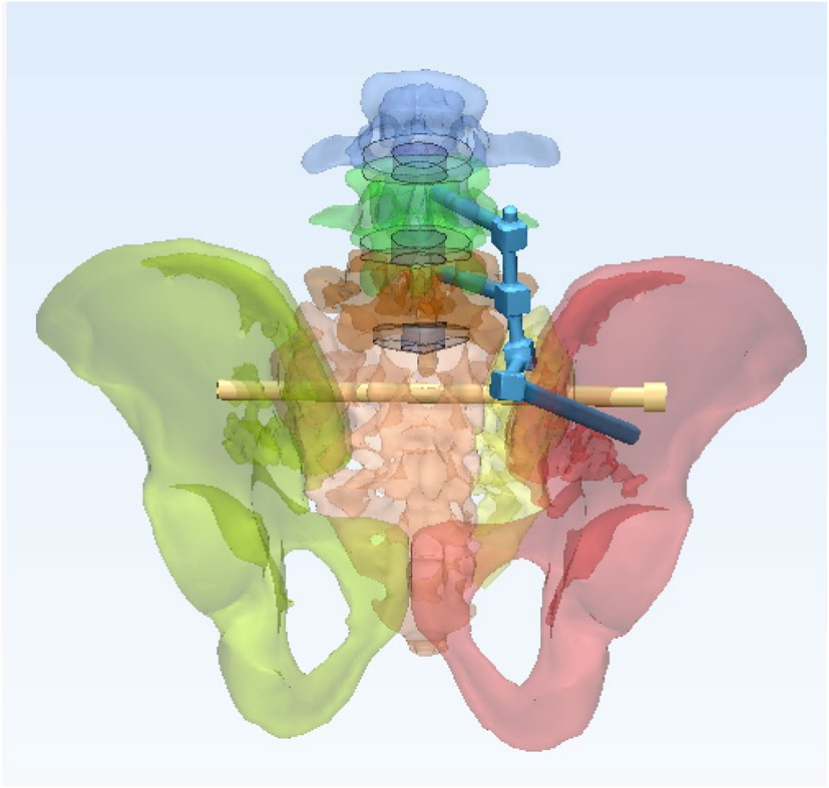
Sketch map of L4L5LS1 (created by Mimics17.0 https://www.materialise.com/en/healthcare/electronic-instructions-for-use/mimics-research).

**Figure 6:**
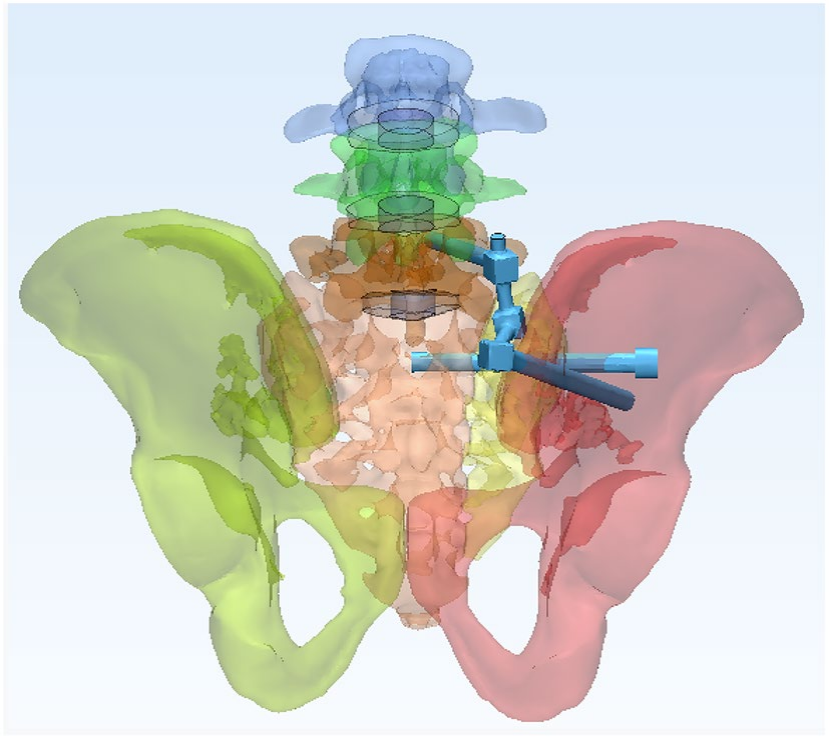
Sketch map of L5NS1 (created by Mimics17.0 https://www.materialise.com/en/healthcare/electronic-instructions-for-use/mimics-research).

**Figure 7:**
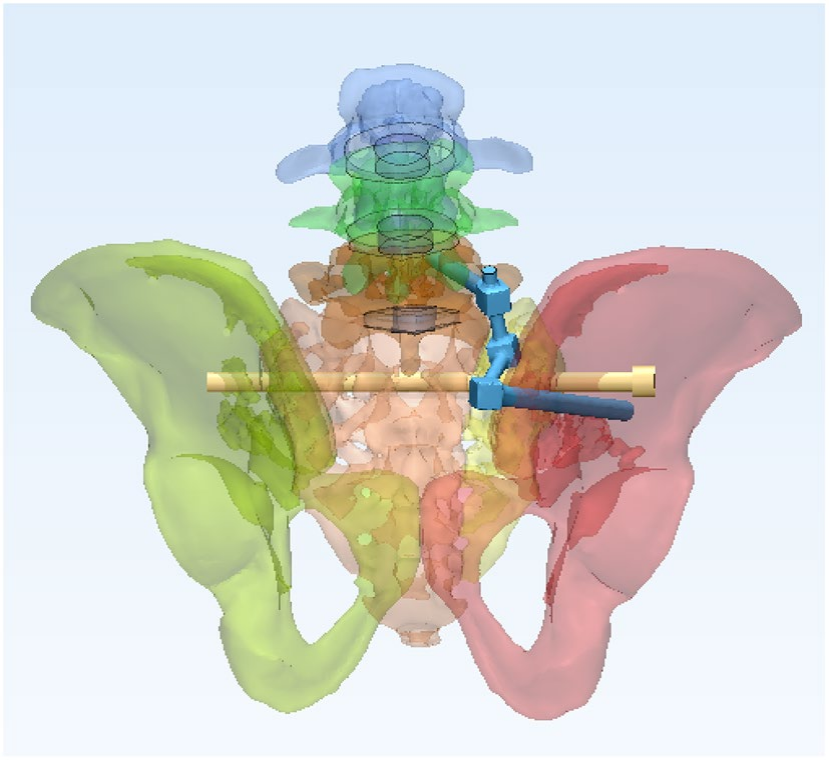
Sketch map of L5LS1 (created by Mimics17.0 https://www.materialise.com/en/healthcare/electronic-instructions-for-use/mimics-research).

In this study, a normal sacroiliac screw was defined as a sacroiliac screw with a length crossing the fracture line to the midline of the sacrum. Lengthened sacroiliac screws were defined as those with a length crossing the fracture line and penetrating the contralateral iliac bone. Four internal fixation models were established in this study (Figs. 4, 5, 6, 7): (1) unilateral L4 + L5 segment iliolumbar fixation + S1 normal sacroiliac screw (L4L5NS1), (2) unilateral L4 + L5 segment iliolumbar fixation + S1 lengthened sacroiliac screw (L4L5LS1), (3) unilateral L5 segment iliolumbar fixation + S1 normal sacroiliac screw (L5NS1), and (4) unilateral L4 + L5 segment iliolumbar fixation + S1 lengthened sacroiliac screw (L5LS1).

The length and diameter of the lumbar pedicle screws and iliac screws were 45 mm and 6.5 mm and 70 mm and 7.5 mm, respectively. The diameter of the sacroiliac screws was 7.3 mm. The length of long screw is 156 mm.The length of normal screw is 78 mm.The material properties were set to titanium alloy.

Boolean operations were performed for the four internal fixation models. The vertical displacements of the above four internal fixation models were recorded and compared with the normal model. Point a and point b were marked on the vertical fracture line of the sacrum (Fig. 2), and two points, a1a2 and b1b2, were generated when the fracture was separated. The distance between these two points was recorded, and the distance was considered the fracture displacement value. The maximum von Mises stress of fixation was recorded, and the cloud of von Mises stress of fixation was analysed to evaluate the stress distribution of internal fixation.

### Ethics approval and consent to participate

This study was approved by the ethics committee of Yantai Shan Hospital and was carried out in accordance with the ethical standards of the Declaration of Helsinki. Informed consent was obtained from each participant included in the study.

## Results

### Sacrum vertical displacement distance

Under a vertical force of 600 N, we recorded the vertical displacement distances of the upper surface of the sacrum for the four fixation patterns. Five points (A, B, C, D, and E) were selected on the upper surface of the sacrum, and the vertical displacement distances of these five points were recorded (Table 3).

**Table 3:** Displacement of the upper surface of the sacrum.

	A (mm)	B (mm)	C (mm)	D (mm)	E (mm
L4L5NS1	0.4025	0.3675	0.3549	0.4256	0.4072
L5NS1	0.397	0.3168	0.3611	0.4375	0.3677
L4L5LS1	0.3095	0.2407	0.2699	0.3256	0.2805
L5LS1	0.3218	0.2527	0.2845	0.3521	0.2937

According to the Shapiro‒Wilk test, the data for all four groups conformed to a normal distribution (Table 4). Analysis of variance performed on the four groups of fixation patterns showed F = 10.44, *p* = 0.0005, indicating that the sacral displacements of the four groups were not all equal (Table 5). The results of two-by-two comparisons suggested that the sacral position displacement in Groups L4L5NS1 and L5NS1 were more significant than those in Groups L4L5LS1 and L5LS1 (*p* < 0.05); the differences between Groups L4L5NS1 and L5NS1, L4L5LS1 and L5LS1 were not statistically significant (*p* > 0.05; Table 6). The results showed that the distance of displacement of the sacrum by the fixation mode used for the lengthened sacroiliac screws was smaller than that of the fixation mode used for the normal sacroiliac screws. The difference between the L4–L5 and L5 fixation modes was not statistically significant.

**Table 4:** Shapiro‒Wilk test.

Variable	Obs	W	V	z	Prob > z
Group = 1: Shapiro–Wilk W test for normal data					
Distance	5	0.92877	0.841	− 0.222	0.58798
Group = 2: Shapiro–Wilk W test for normal data					
Distance	5	0.98529	0.174	− 1.760	0.96078
Group = 3: Shapiro–Wilk W test for normal data					
Distance	5	0.97646	0.278	− 1.371	0.91487
Group = 4: Shapiro–Wilk W test for normal data					
Distance	5	0.98824	0.139	− 1.929	0.97315

Group 1 L4L5NS1, Group 2 L5NS1, Group 3 L4L5LS1, Group 4 L5LS1.

**Table 5:** Analysis of variance.

	Summary of distance				
Group	Mean	Std. dev.	Freq.		
1	0.39154	0.02935069	5		
2	0.37602	0.04477385	5		
3	0.28524	0.03338814	5		
4	0.30096	0.0377567	5		
Total	0.33844	0.05802008	20		

	Analysis of variance				
Source	SS	df	MS	F	Prob > F
Between groups	0.042334285	3	0.014111428	10.44	0.0005
Within groups	0.021625985	16	0.001351624		
Total	0.06396027	19	0.00336633		
Bartlett’s equal-variances test: chi2(3) = 0.7087 Prob > chi2 = 0.871					

Group 1 L4L5NS1, Group 2 L5NS1, Group 3 L4L5LS1,Group 4 L5LS1.

**Table 6:** Tukey HSD test.

Grp versus grp	Group Means	Group Means	Mean dif	HSD-test
1 versus 2	0.3915	0.3760	0.0155	0.9439
1 versus 3	0.3915	0.2852	0.1063	6.4653*
1 versus 4	0.3915	0.3010	0.0906	5.5092*
2 versus 3	0.3760	0.2852	0.0908	5.5214*
2 versus 4	0.3760	0.3010	0.0751	4.5653*
3 versus 4	0.2852	0.3010	0.0157	0.9561

Tukey HSD pairwise comparisons for variable group studentized range critical value (0.05, 4**,** 16) = 4.0460967 uses harmonic mean sample size = 5.000.

Group 1 L4L5NS1, Group 2 L5NS1, Group 3 L4L5LS1, Group 4 L5LS1.

### Fracture separation value

**Figure 8:**
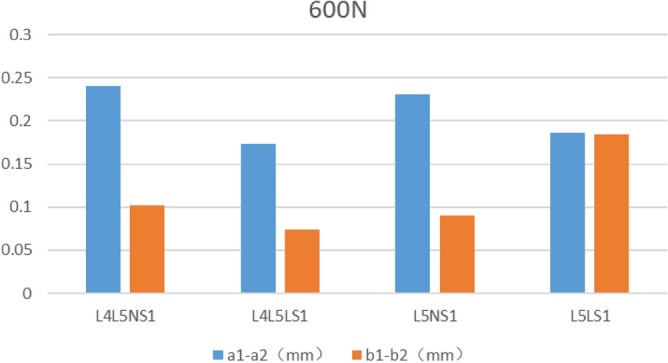
Fracture separation value under 600N vertical load.

**Figure 9:**
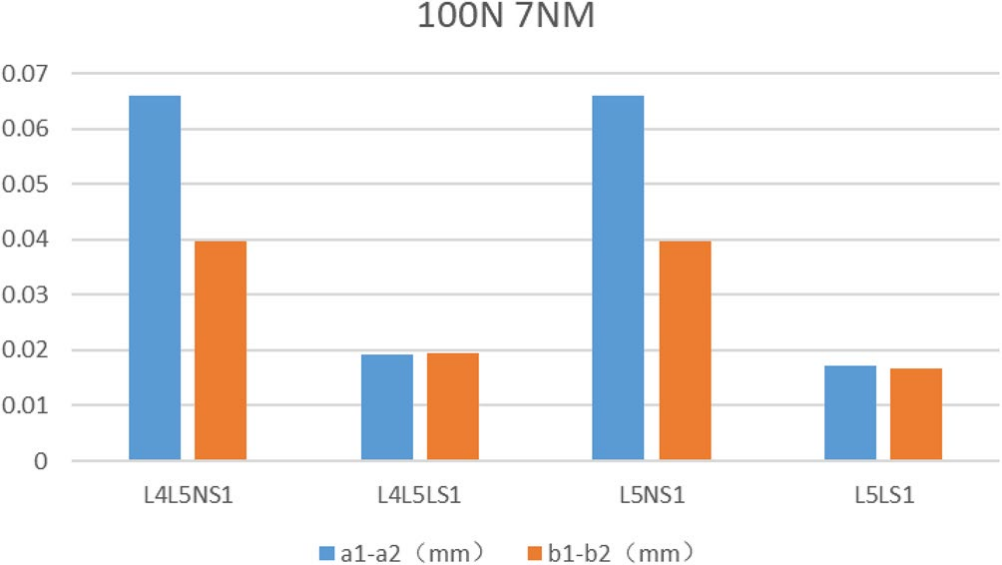
Fracture separation value under 100N slave load and 7Nm torque.

The fracture separation values of the four fixed models were recorded under 600 N vertical pressure (Table 7 and Fig. 8). Comparing the a1–a2 values, the minimum value of L4L5LS1 was 0.1738 mm. The maximum value of L4L5NS1 was 0.241 mm. Comparing the b1–b2 values, the minimum value of L4L5LS1 was 0.074 mm, and the maximum value of L5LS1 was 0.1844 mm. The bone seam separation values were recorded for the four fixation models under 100 N of a slave load and 7 Nm torque (Table 7, Fig. 9). Comparing the a1–a2 distance, L5LS1 had a minimum value of 0.017 mm, followed by L4L5LS1 with 0.019 mm. L4L5NS1 had a maximum value of 0.08 mm. Comparing the b1–b2 distance, L5LS1 had a minimum value of 0.0168 mm, followed by L4L5LS1 with 0.0194 mm; L4L5NS1 had a maximum value of 0.0397 mm.

**Table 7:** Experimental results in each simulation state.

	600N			100N 7NM		
	a1–a2	b1–b2	Maximum von Mises stress (MPa)	a1–a2	b1–b2	Maximum von Mises stress (MPa)
L4L5NS1	0.241	0.102	131.1	0.0659	0.0397	87.18
L4L5LS1	0.1738	0.074	107.9	0.0191	0.0194	42.65
L5NS1	0.2307	0.09	111	0.0659	0.0397	44.83
L5LS1	0.186	0.184	112.2	0.0171	0.0168	40.8

### The von Mises stress

**Figure 10:**
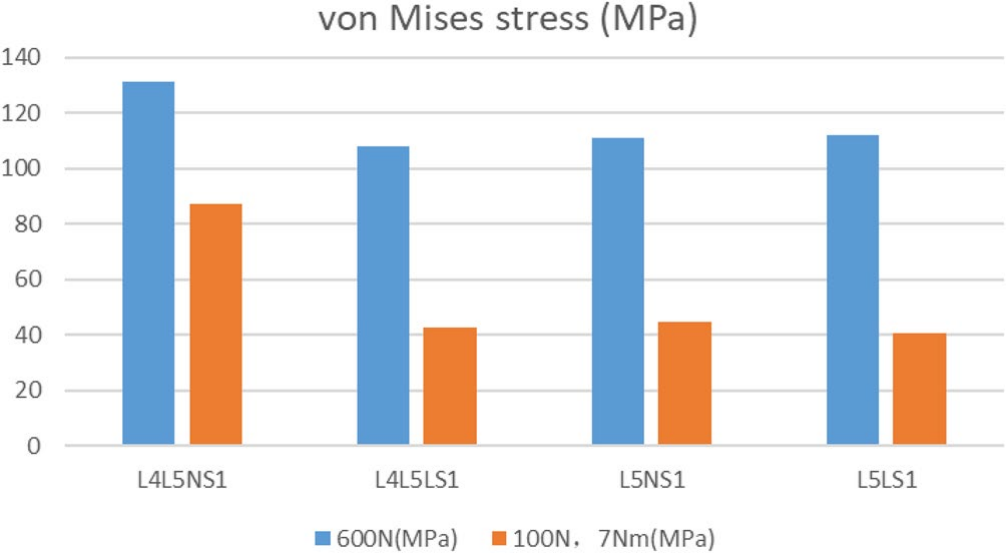
The Maximum von Mises stress.

The maximum von Mises stress of the implant was recorded (Fig. 10 and Table 7). The maximum von Mises stress of L4L5NS1 was the largest, at 131 MPa under a vertical force of 600 N. The other three models were close in value. The maximum von Mises stress of L4L5NS1 was the largest, at 87.1 MPa under a slave load of 100 N and torque of 7 Nm. The other three groups of models were close in value. Analysing the von Mises stress distribution of the four groups of internal fixation models, triangular fixation under a vertical load of 600 N showed the stresses to be concentrated around the fracture ends of the linked pedicle screws and iliac screws, as well as the sacroiliac screws. Analysing the von Mises stress distribution of the four groups of internal fixation models, stress concentrations were observed at the pedicle screw and iliac screw attachment bar and around the sacroiliac screw fracture under a vertical load of 600 N. Under a 100 N slave load + 7 Nm torque, stress concentrations were observed at the pedicle screw, the pedicle connecting rod, the iliopsoas screw connecting rod, and the sacroiliac screw fracture.

## Discussions

The sacrum is an essential component of the pelvic ring, and unstable sacral fractures severely affect the integrity and stability of the posterior pelvic ring, leading to traumatic spine-pelvis separation, and poor fracture repositioning can affect body weight-bearing and lower-limb function. Treatment of unstable sacral fractures aims to rebuild the stability of the spine and pelvis, restore the biomechanical conduction of the lower extremity-pelvis-spine, and simultaneously achieve nerve decompression when combined with nerve injury. The traditional posterior fixation methods commonly used in clinical practice include sacral rod fixation, posterior tension band plate fixation, and sacroiliac screw fixation. Sacroiliac screws and iliolumbar fixation have been most commonly used.

The advantage of iliolumbar fixation is mainly in reconstructing the spine in the vertical direction. In 1994, Kach and Trentz^22^ first reported the successful treatment of longitudinally displaced sacral fractures using pedicle nailing and interiliac crest bracing, introducing the concept of the spine-pelvis bracing technique. We achieved the lumbar-pelvic fixation by connecting the L4 and L5 pedicle screws to the iliac crest screws with a rod. This technique is effective against vertical pelvic instability because it fixes the lumbar spine and pelvis with an arch nail system and has a bracing and closing effect, which is vertical. When there is concurrent sacral nerve injury and sacral canal occupancy, posterior exploration can be performed for decompression and nerve repair simultaneously. This technique applies to all vertically unstable pelvic fractures. However, there are inherent disadvantages of this fixation method, as follows: a sizeable surgical incision, which may cause complications such as infection and nonunion; slightly less effective fixation for unstable transverse fractures, which may cause fracture line separation; restriction of lower lumbar movement, which may cause scoliosis due to fixation on one side; the need to remove the internal fixation after fracture healing; and the need to bend the connecting rod, which increases the difficulty of fixation. Sacroiliac screw fixation is a significant advance in treating unstable sacral fractures and has become a minimally invasive technique commonly used to treat these fractures. These are the advantages of sacroiliac screws, such as minimal surgical injury, low rate of postoperative infection, and minimal blood loss^23,24^.Compared with other posterior internal fixation techniques, the incidence of vascular and nerve injury caused by sacroiliac screws is higher, approximately 2–15%^25^. Kraemer et al.^26^ compared the extraction force of sacral body long screws, sacral body short screws, and sacral wing short screws, with means of 925 N, 327 N, and 71 N, respectively, and the differences were statistically significant. Sacroiliac screws that have been lengthened are utilized to strengthen the stability of the sacral fracture. Gardner and Routt^27^ proposed lengthened sacroiliac screws. The screws penetrate from the sacroiliac joint on one side to the sacroiliac joint on the other, achieving adequate stability. Jazini et al.^28^ concluded that vertical shear is the primary stress-causing instability of the posterior pelvic ring, confirming through biomechanical tests that this stress is distributed over the entire screw. Therefore, one longer screw allows for a more reasonable distribution of stresses^29^. The most extended screw that spans the entire sacroiliac complex is the lengthened sacroiliac screw, which is particularly suitable for bilateral sacral fractures. The number of cortical bones crossed medially and laterally by the lengthened sacroiliac screws is essentially the same at the fracture line, providing a balanced fixation. The lengthened sacroiliac screws used in the present study penetrate the contralateral cortex. Nevertheless, sacroiliac screws have some shortcomings. Keating et al.^30^ applied sacroiliac screws and achieved 84% anatomic or near-anatomic reduction of pelvic fractures, but the healing rate of the deformity at follow-up was as high as 44%.

Griffin et al.^8^ concluded that sacroiliac screw fixation of vertical sacral fractures may result in internal fixation failure and loss of reduction.

The strength of iliolumbar fixation and sacroiliac screw fixation is not sufficient. Schildhauer et al.^12^ proposed triangular osteosynthesis, a vertically oriented spinal pelvic fixation combined with a transverse fixation device. The biomechanical study by Schildhauer et al.^31^ also showed that triangular fixation was stronger than sacroiliac screw fixation for unstable sacral fractures. As there are still many questions about the biomechanical properties of triangular fixation that need to be addressed, we performed a finite element biomechanical study of triangular osteosynthesis.

This study modelled a finite element model with a longitudinal cut through the right sacral foramen to create a unilateral vertical sacral fracture model (AO C3.1 Denis II). Unilateral vertical sacral fractures involving the L5/S1 tuberosity are often exceedingly unstable; however, in this case, the model was simplified, and the fracture line did not involve the L5/S1 tuberosity. In the fixation model, sacroiliac screws were used for trans-S1 segmental fixation, with normal sacroiliac screws and lengthened sacroiliac screws. The effect of increasing the length of sacroiliac screws on the biomechanical properties of internal fixation with triangular fixation was then evaluated. The design of the iliolumbar fixation model took into account that the fracture model involved unilateral sacral vertical fracture using a unilateral iliolumbar fixation model. This study used two L4–L5 segments or a single L5 segment for lumbar fixation, and whether increasing the fixation segment affects the biomechanical properties of internal fixation was assessed.

The sacral vertical displacement distance is an important index for determining the vertical stability of the sacrum. Under a vertical load of 600 N, the vertical displacement distance of the normal sacral model in this study was 0.159 mm. None of the four fixation models could achieve stability of the sacrum in the normal state under fixation. L5LS1 and L4L5LS1 showed the best vertical stability among the four fixation models, and the results were not significantly different between these two groups. We found that the fixation model achieved the best state of sacral stability with lengthened sacroiliac screws. Therefore, increasing the length of sacroiliac screws can increase the vertical stability of the sacrum when applying the triangular fixation technique to treat unilateral vertical sacral fractures. Overall, there was no significant difference in the vertical stability of the sacrum between L4–L5 fixation and L5 fixation in either the normal sacroiliac screw group compared with the other groups or in the lengthened sacroiliac screw group compared with the other groups. Moreover, increasing the fixed segment of the lumbar spine did not increase the stability of the triangular fixation device. This phenomenon may be because increasing the lumbar fixation segments alters the normal force transmission in the lumbar spine. The finding that increasing the length of the sacroiliac screw increased the vertical stability of the sacrum is consistent with the literature^18^. The vertical displacement of the sacrum was not significant at 100 N follower load + 7 Nm torque.

The fracture separation distance represents the degree of stability of the fracture line in the fixed state. Under a vertical load of 600 N, the superior fracture line displacement distance was more significant than the inferior one. This phenomenon is consistent with the biomechanical characteristics of the pelvis. As the sacrum is under vertical force and the force is transmitted along the sacroiliac joint-pelvis-acetabulum, the closer the fracture line is to the mechanical transmission path, the greater is the displacement. Comparing the a1–a2 distance in the four fixation models under 600 N vertical load and 100 N slave load + 7 Nm torque, the fracture separation distance with lengthened sacroiliac screws was significantly smaller than that in the model with normal sacroiliac screw fixation. However, in the same sacroiliac screw model, there was no significant difference in the a1–a2 distance between the models with the L4–L5 lumbar fixation segment and L5 segment. Increasing the length of the sacroiliac screw when applying the triangular fixation technique to fix unilateral vertical sacral fractures increased the stability of the fracture end, but increasing the lumbar fixation segment had no significant effect on fracture stability.

The implant von Mises stress represents the implant stress state in the finite element model. This study compared four groups of the implant model's maximum von Mises stress. The maximum von Mises stress value of L4L5LS1 was the smallest, at 107.9 MPa under a 600 N vertical load. The maximum von Mises stress value of L4L5NS1 was the largest, at 131 MPa. Under 100 N + 7 Nm load, the maximum von Mises value of the L5LS1 model was a minimum of 40.8 MPa, and the maximum von Mises value of L4L5NS1 was 87.18 MPa. Therefore, the internal fixation stress of the fixed L4–L5 plus S1 lengthened sacroiliac screw combination was minor, regardless of the standing condition or the lumbar rotation condition. The maximum von Mises values of the four fixation models in this study did not differ significantly with motion, except for L4L5NS1. This result suggests alteration of the normal mechanical conduction of the lumbar spine after fixation of the L4–L5 segment. Long segment fixation of the lumbar spine alters the mechanical conduction of the lumbar spine, and the application of normal screw fixation increases the internal fixation stress concentration. By analysing the von Mises stress distribution of the four groups of internal fixation models, with triangular fixation under a vertical load of 600 N, the stresses were concentrated around the fracture ends of the linked pedicle screws and iliac screws, as well as the sacroiliac screws. Based on the von Mises stress distribution of the four groups of internal fixation models, stress concentrations were observed at the pedicle screw and iliac screw attachment bar and around the sacroiliac screw fracture under a vertical load of 600 N. Under a 100 N slave load + 7 Nm torque, stress concentrations were observed at the pedicle screw, the pedicle connecting rod, the iliopsoas screw connecting rod, and the sacroiliac screw fracture. This result is also consistent with clinical practice.

It is essential to note that this study has some limitations. Some patients have anatomical variants of the sacrum, without lengthened sacroiliac screw channels. This is commonly seen in the S1 segment. Indeed, lengthened sacroiliac screws are not suitable for all patients, and preoperative CT evaluation is exceptionally important. This study used a unilateral vertical sacral fracture (AO C3.1 Denis II) type, and the sacrum was cut longitudinally to create a vertical sacral fracture model. However, because of the variety of anterior pelvic ring injuries, treatment methods are equally diverse, and the different treatment methods undoubtedly impacted the results of this study. In addition, the increase in influencing factors inevitably increased the difficulty of data analysis. This study was not designed to address anterior ring injuries, preserving the integrity of the anterior ring. This study is a finite element study based on pelvic CT data. Although finite element studies have made significant progress in recent years, there may be some differences between this type of study and human studies.

Using iliolumbar fixation combined with sacroiliac screws for unilateral vertical sacral fractures (AO C3.1 DENISII), the application of lengthened sacroiliac screws increased the vertical stability of the sacrum after internal fixation. The use of triangular fixation, with simultaneous fixation of the L4 and L5 segments, had no significant effect on the increase in vertical stability of the sacral fracture. Fixation of only the L5 segment reduces the complications of multi-segment lumbar fixation and is not biomechanically inferior. We should try to use the L5 segment for lumbar fixation.

## Data Availability

The datasets used and analyzed during the current study are available from the corresponding author on reasonable request.
